# Mediating Role of Psychological Capital in the Relationship Between Social Support and Self-Neglect Among Chinese Community-Dwelling Older Adults

**DOI:** 10.3389/fpsyg.2022.903625

**Published:** 2022-06-22

**Authors:** Binyu Zhao, Hangsai Wang, Chunqi Xie, Xianhong Huang, Meijuan Cao

**Affiliations:** ^1^School of Nursing, Hangzhou Normal University, Hangzhou, China; ^2^School of Medicine, Jinhua Polytechnic, Jinhua, China; ^3^Department of Health Policy and Management, School of Public Health, Hangzhou Normal University, Hangzhou, China

**Keywords:** self-neglect, social support, psychological capital, older adults, mental health

## Abstract

**Objectives:**

Self-neglect in older adults has become an important public health issue and is associated with negative health outcomes and increased morbidity and mortality. Social support has been recognized as a prominent predictor of self-neglect, but the underlying mechanism is unclear. This study aims to investigate and illustrate the associations among social support, psychological capital, and self-neglect.

**Methods:**

This study used a cross-sectional convenience sampling design. A total of 511 older adults were recruited in Chinese communities. Spearman’s correlation coefficient and hierarchical multiple regression analysis were performed to assess the influencing factors of self-neglect. A structural equation model was applied to test the hypothesized mediation model.

**Results:**

Social support and psychological capital were found to be negatively related to self-neglect. Social support and psychological capital explained 5.1 and 11.9% of the incremental variances of older adults’ self-neglect, respectively. Psychological capital acts as a mediator between social support and self-neglect.

**Conclusion:**

Self-neglect among older adults is a rising problem in China. Social support and higher psychological capital could decrease the risk of self-neglect in older adults. It is crucial to improve social networks and facilitate psychological interventions to reduce such self-neglect.

## Introduction

The Chinese population is aging at an unprecedented pace, and older population aged over 65 years will reach 336 million by 2050, accounting for one-third of the total Chinese population (National Bureau of Statistics of China, 2017; Zhong et al., 2018). Alongside the increased aging population, self-neglect is becoming a prominent problem that challenges healthy aging (Cipriani et al., 2021; Yu et al., 2021).

Self-neglect, the result of complex interactions between social, psychological, and medical factors, refers to older adults’ neglect of their personal hygiene, health, and living environment either intentionally or unintentionally, and their refusal or failure to engage in self-care behaviors, which threatens their own health and safety (Dong, 2014; Touza and Prado, 2019; Wang et al., 2021). Evidence has suggested that self-neglect among older adults can induce negative outcomes, including nonadherence to medication, multiple forms of abuse, and impairment of cognitive function and physical function (Dong et al., 2009a, 2013; Dong and Simon, 2013). According to Papaioannou et al. (2012), self-neglect can also lead to malnutrition, frailty, and the deterioration of physical ability among older people, thus increasing their risk of falls and fractures. Furthermore, self-neglect has been identified as an independent risk factor for mortality among older adults. A large-scale prospective research, investigating self-neglect in 9,318 community-dwelling older adults with a follow-up period of over 5 years, revealed that the 1-year mortality of older adults who were diagnosed with self-neglect was 5.87 times that of older adults who were not thus diagnosed (Dong et al., 2009b). It has also been reported that self-neglect is significantly associated with an increased risk of short-term, long-term, and lifetime suicidal ideation among older adults (Dong et al., 2017; Yu et al., 2019). In China, the prevalence of self-neglect among older adults has reached 23.2% (Yu et al., 2019), whereas its incidence rate is only 8.4% in Chicago (Wang et al., 2020) and 11.5% in Iran (Mohseni et al., 2019). Chinese culture, a collectivist culture, influence many older adults to devote all their time and energy to their offspring rather than to their own well-being; this might offer an explanation for the high incidence of self-neglect (Wu et al., 2020). Furthermore, Chinese older adults consider self-neglect normal (Wu et al., 2020), which might lead to them refusing relative interventions. However, *Health China Action* (2019–2030), released by the Chinese government in 2019, emphasizes the importance of improving mental health among older adults. It encourages family members to concern themselves with the psychological state of older adults in the family, and encourages communities to carry out activities to provide psychological support (National Health Commission of China, 2019). Therefore, it is necessary to investigate the precipitating factors and root causes of self-neglect in the Chinese context to develop effective and alternative interventions.

From the perspective of sociology, multiple theoretical models have indicated that social support, a type of social capital, is a prominent predictor of self-neglect among older adults (Dyer et al., 2007; Iris et al., 2010). For instance, conceptual model proposed by Dyer states that a lack of social support would lead to inadequate support services (such as medical care and assistance with bathing, dressing, home-cleaning, laundry, and procuring/preparing food), which ultimately results in self-neglect among older adults (Dyer et al., 2007). In Iris et al. (2010) model, social network is considered an important and independent risk factor for self-neglect among older adults (Iris et al., 2010). Additionally, several studies report a significant association between social support and self-neglect in different countries (Burnett et al., 2006; Dyer and Reyes-Ortiz, 2017). However, the implementation of the one child policy, changed family structures, and the large-scale migration of younger adults seeking employment has decreased the social support for older adults in China (Cao et al., 2015; Zhong et al., 2018; Gao and Cheng, 2020). Therefore, there is an urgent need to find alternative solutions. Meanwhile, exploring the underlying mechanism between social support and self-neglect at the psychological level might provide a more comprehensive theoretical basis for alleviating self-neglect.

Psychological capital (PsyCap) refers to a positive psychological state of personal development (Minglu et al., 2020; Chen and Yitao, 2021; Gu et al., 2021). It stems from positive psychology, which is an emerging area in psychology and holds the view that positivity is one of the remedies to negative social and psychological indicators, such as self-neglect (Hefferon and Boniwell, 2011; Pluskota, 2014; Gu et al., 2021). The risk and vulnerability model of self-neglect indicates that psychological status impacts older adults’ vulnerability to self-neglect (Paveza et al., 2008). Furthermore, four key features of PsyCap—self-efficacy, optimism, hope, and resilience—have been shown to have a negative association with self-neglect. For example, self-efficacy is associated with self-neglect and mediates the relationship between self-neglect and related factors such as functional dependency and social networks (Dahl et al., 2020). Optimism and hope are reported to be negative predictors of self-neglect, and resilience is considered an important factor for reducing the risk of self-neglect among older adults (Gunstone, 2003; Genke, 2004; Minayo et al., 2019). A qualitative study demonstrated that self-realization is part of the understanding of PsyCap from the perspective of Chinese older adults that is recognized and appreciated by Chinese culture (Shi, 2013) and might increase older adults’ acceptance of interventions and enhance their resistance to self-neglect. Meanwhile, PsyCap can be converted from an external social support to an internal resource (Gu et al., 2021). Studies report that PsyCap mediates the relationship between social support and loneliness (Luthans et al., 2007). Additionally, an increasing number of studies suggest that the components of PsyCap—self-efficacy, optimism, and resilience—can be predicted by social support (Labrague and De Los Santos, 2020; Jemini-Gashi et al., 2021; Sagi et al., 2021). Therefore, we hypothesized that PsyCap mediates the relationship between social support and self-neglect.

Although an increasing number of studies pay close attention to the exploration of the mechanisms of self-neglect from a sociological view, the role of psychology in the relationship between social support and self-neglect remains to be investigated. Therefore, the present study aims to measure social support, PsyCap, and self-neglect among Chinese community-dwelling older adults and to explore the potential relationships among these variables, which might enrich the existing theoretical model and offer a breakthrough for future interventions.

*Hypothesis 1*: Social support is negatively associated with self-neglect.

*Hypothesis 2*: PsyCap is negatively associated with self-neglect.

*Hypothesis 3*: PsyCap mediates the relationship between social support and self-neglect.

## Materials and Methods

### Study Sample and Data Collection

The present cross-sectional study was carried out between October 2017 and January 2018 in the Zhejiang Province, using a simple random sampling method. Older people who (i) were permanent residents; (ii) were ≥ 65 years old, (iii) were able to communicate in Chinese and express themselves clearly, and (iv) consented to participate in the study were included. Older people who (i) had a history of cognitive, psychiatric, or neurological disorder in electronic health records or (ii) had severe diseases were excluded. This study was conducted by four nursing graduates who had adequate experience in site survey. Before data collection, the investigators received uniform training in questionnaire administration and interviewing. With the assistance of local contact people, including community leaders, health workers, or community nurses, the investigators performed household visits to recruit the participants. During data collection, face-to-face interviews were conducted. The investigators offered a detailed explanation of the study to the participants, including the aim and procedures, and obtained their written consent. Questionnaires were completed by the participants themselves, and assistance was provided whenever they did not understand any of the items. The questionnaires were collected and examined on the spot. Manual double entry of survey data was adopted to minimize data-entry errors (Zhong et al., 2018; Wang et al., 2021). Overall, 533 older adults responded to the survey (effective response rate: 95.9%); of these, 511 questionnaires were valid.

### Measures

#### Sociodemographic and Clinical Characteristics

Based on the literature, the survey questionnaire examined the following sociodemographic characteristics: age, sex, marital status, religious beliefs, educational level, monthly income, living arrangement, health insurance, and presence of chronic illness (es; San Filippo et al., 2007; Papaioannou et al., 2012; Yu et al., 2021).

#### Self-Neglect

Self-neglect was measured using the Elder Self-Neglect Assessment (ESNA), which was developed by Iris and translated into Chinese by Wang (Iris et al., 2014; Wang, 2018). While the original ESNA was a 25-item short form consisting of two dimensions (behavioral characteristics and environmental factors; Iris et al., 2014), its Chinese adaptation is a 24-item form measuring three dimensions (lifestyle/condition, health care, and living environment; Wang, 2018). The items are scored as follows: 0 = “no (problem does not exist),” “not applicable,” or “do not know”; 1 = “suspected problem” and; 2 = “yes (problem exists).” Cronbach’s alpha for the ESNA in this study was 0.918; additionally, the Cronbach’s alpha coefficient for each dimension ranged from 0.803 to 0.884, and the test–retest reliability was 0.893.

#### PsyCap

PsyCap was assessed using the Geriatric Psychological Capital Scale, which was developed by Shi (2013) and is widely used in China for measuring geriatric PsyCap. The scale consists of 4 dimensions—self-efficacy, diligence and adamancy, honesty and steadiness, and gratitude and dedication—assessed using 20 items such as “suffering strengthens me” and “I understand my own worth.” A five-point Likert scale ranging from 1 to 5, where 1 = “totally disagree,” 2 = “mostly disagree,” 3 = “not sure,” 4 = “mostly agree,” and 5 = “totally agree,” was used to rate each item. A higher score indicates a higher level of PsyCap. Cronbach’s alpha for this scale was 0.935 in the present study.

#### Social Support

Social support was assessed using the Social Support Revalued Scale developed by Xiao, which has been used extensively in research conducted in China (Ren et al., 2020; Zhan et al., 2020). It consists of 10 items and measures subjective support, objective support, and support availability (Geng et al., 2017). Items assess the support received from friends, neighbors, families, colleagues, and the society. The cumulative score ranges from 12 to 66 points, with higher scores indicating higher levels of social support. The Cronbach’s alpha for this scale was previously found to be 0.896 (Xiao, 1994). In this study, the Cronbach’s alpha for the scale was 0.799.

### Ethical Considerations

This study was approved by the Ethics Committee of Hangzhou Normal University (approval number: 2022014). Written or verbal informed consent was provided by every participant, each of whom was informed of the aim of the research, its significance, and the data collection processes. In this process, the researcher explained the participants’ right to refuse or withdraw at any time during the survey interview.

### Statistical Analysis

Prior to analyses, normality, outliers, and multicollinearity were assessed. Normality was tested using skewness(sk) and kurtosis(ku), and values fell within the acceptable range (sk < |3|; ku < |10|; Kline, 2015). The Cook’s distance was applied to identify the outliers. The maximum Cook’s distance was <0.5, indicating that no outliers existed in this data (Huang et al., 2022). Multicollinearity was tested by variance inflation factor (VIF), and the finding showed no VIF > 10, indicating the absence of multicollinearity.

The relationship between older adults’ self-neglect and sociodemographic variables was analyzed with t-tests and one-way ANOVA using SPSS version 25 (IBM, Armonk, New York, Unite States). Spearman’s correlation analysis was applied to explore the associations among self-neglect, social support, and PsyCap. Hierarchical multiple regression analyses (HMR) were conducted to test the factors influencing older adults’ self-neglect and their contribution toward predicting self-neglect, which comprised—Step 1: participant’s sociodemographic characteristics; Step 2: participant’s social support; and Step 3: participant’s PsyCap. Standardized parameter estimates (*β*) were performed to evaluate the magnitudes of associations among social support, PsyCap, and self-neglect. The mediation pathways of PsyCap between social support and self-neglect were analyzed using SPSS Amos 26.0 (IBM Corp., Armonk, NY, United States). When the value of *χ^2^*/df (degree of freedom) < 5; goodness of fit index (GFI), adjusted GFI (AGFI), incremental fit index IFI, and Tucker–Lewis index (TLI) > 0.90; and root mean square error of approximation (RMSEA) < 0.08, the hypothesized model was considered to be a close fit of the data (Bagozzi and Yi, 1988; Joreskong and Sorbom, 1993). To estimate the indirect effect, bootstrapping was applied due to non-normal distribution variables in the sample (Shrout and Bolger, 2002). Indirect effects were assessed with a 95% confidence interval (95%CI) of indirect effects in an empirical sampling distribution. If the 95% CI was nonzero, the indirect effect was considered significant. Statistical significance was set as *p <* 0.05.

## Results

### Sociodemographic Characteristics of Participants

The mean scores for older adults’ self-neglect, social support, and PsyCap were 13.65 ± 10.65, 35.89 ± 8.90, and 74.85 ± 15.51, respectively, which were approximately 28.4, 54.4, and 74.9% of the total score, respectively. Table 1 shows participants’ sociodemographic characteristics and the distribution of self-neglect. A total of 511 older adults participated in the study. The mean age (±SD) of the participants was 76.14 ± 6.99 years, and 53.62% were women. The percentages of older people living with a spouse and children, with a spouse, with children, alone, and with others were 12.13, 58.12, 9.39, 19.57, and 0.78%, respectively.

**Table 1:** Sociodemographic characteristics of the participants (*N* = 511).

**Variable**	***N* (%)**	**Self-neglect Mean (SD)**	***P***	**Variable**	***N* (%)**	**Self-neglect Mean (SD)**	***P***
Age (years)			**0.035**	0–999	247 (48.34)	20.53 (9.974)	
60–69	112 (21.92)	13.09 (7.531)		1,000–1999	56 (10.96)	12.95 (6.454)	
70–79	214 (41.88)	13.02 (10.362)		2000–2,999	55 (10.76)	11.36 (6.337)	
80–89	169 (33.07)	14.15 (11.919)		3,000–3,999	65 (12.72)	4.34 (3.332)	
90–99	16 (3.13)	20.75 (15.931)		≥4,000	88 (17.22)	3.10 (2.905)	
Sex			**0.151**	Living arrangement			**<0.001**
Male	237 (46.38)	12.92 (9.783)		Living with spouse and children	62 (12.13)	13.82 (10.519)	
Female	274 (53.62)	14.28 (11.321)		Living with spouse	297 (58.12)	12.23 (9.846)	
Marital status			**<0.001**	Living with children	48 (9.39)	12.31 (7.066)	
Married	364 (71.2)	12.52 (9.945)		Living alone	100 (19.57)	18.95 (12.644)	
Widowed, divorced, and other	147 (28.8)	16.46(11.785)		Living with others	4 (0.78)	0.50 (1.000)	
Religious beliefs			**<0.001**	Health insurance			**0.194**
Have	154 (30.14)	16.73 (10.724)		Have	503 (98.43)	13.57 (10.483)	
Do not have	357 (69.86)	12.32 (10.348)		Do not have	8 (1.56)	18.5 (18.701)	
Educational level			**<0.001**	Presence of chronic illness(es)			**0.152**
Primary school and below	366 (71.62)	17.28 (10.139)		0	66 (13.11)	13.18 (9.067)	
Junior high school	58 (11.35)	6.17 (5.663)		1	215 (42.07)	13.13 (10.444)	
Senior high school	55 (10.76)	4.07 (4.350)		2	135 (26.42)	13.13 (10.914)	
Training school and above	32 (6.26)	2.13 (1.50)		≥3	95 (18.40)	15.92 (11.561)	
**Monthly income (RMB)**			**<0.001**				

SD: Standard Deviation, 1RMB=US $0.15.

### Associations Among Self-Neglect, Social Support, and PsyCap

As shown in Table 2, both social support (*r* = −0.638, *p* < 0.01) and PsyCap (*r* = −0.812, *p* < 0.01) were negatively associated with self-neglect in older adults, revealing that they were suitable for further hierarchical linear regression analysis and SEM. At the same time, social support was positively associated with PsyCap (*r* = 0.674, *p* < 0.01).

**Table 2:** The correlation between self-neglect, social support, and psychological capital.

S. No.		1	2	3
1.	Self-neglect	1		
2.	Social support	−0.638^**^	1	
3.	Psychological capital	−0.812^**^	0.674^**^	1

** significant at the 0.01 level (two-tailed).

### Hierarchical Linear Regression Analysis of Self-Neglect

Table 3 shows the result of HMR models for older adults’ self-neglect. Older age was positively associated with self-neglect (*p* < 0.01). Additionally, higher monthly income (1000–1999, 2000–2,999 yuan) was negatively associated with self-neglect (*p* < 0.05) compared with lower income (0–999 yuan). Social support was significantly and negatively associated with self-neglect, contributing to 5.1% of the variance. PsyCap was also significantly and negatively associated with self-neglect, explaining for an additional 11.9% of the variance. The regression coefficient (*β*) for the association between social support and self-neglect was reduced from 0.323 to 0.153 when PsyCap was added to the model, indicating that PsyCap might partially mediate the effect of social support on self-neglect among Chinese community-dwelling older adults.

**Table 3:** The hierarchical multiple regression analysis of elder self-neglect (N = 511).

**Variable**	**Model 1**			**Model 2**			**Model 3**		
	***β***	**Standardized *β***	**95%CI**	***β***	**Standardized *β***	**95%CI**	***β***	**Standardized *β***	**95%CI**
**Sociodemographic characteristics of the participants**									
Age (years)	0.154^**^	0.104^**^	0.055 to 0.252	0.136^**^	0.092^**^	0.043 to 0.228	0.127^**^	0.086^**^	0.048 to 0.205
Sex	−0.192	−0.009	−1.552 to 1.169	−0.214	−0.010	−1.499 to 1.071	−0.689	−0.032	−1.776 to 0.399
Marital status	−3.842	−0.164	−9.763 to 2.078	−2.971	−0.126	−8.566 to 2.624	−0.131	−0.006	−4.875 to 4.612
Religious beliefs	−0.341	−0.015	−1.833 to 1.150	0.186	0.008	−1.229 to 1.601	0.704	0.030	−0.494 to 1.901
**Educational level**									
(reference category: Primary school and below)									
Junior high school	−0.875	−0.026	−3.538 to 1.788	−0.846	−0.025	−3.360 to 1.669	−0.324	−0.010	−2.450 to 1.802
Senior high school	−1.949	−0.057	−4.900 to 1.002	−2.093	−0.061	−4.880 to 0.695	−1.152	−0.034	−3.510 to 1.207
Training school and above	−3.084	−0.07	−6.768 to 0.600	−2.950	−0.067	−6.429 to 0.529	−0.298	−0.007	−3.260 to 2.664
**Monthly income**									
(reference category: 0–999 RMB)									
1,000–1999	−6.739^***^	−0.198^***^	−8.942 to −4.536	−5.525^***^	−0.162^***^	−7.627 to −3.422	−2.207^*^	−0.065^*^	−4.043 to −0.372
2000–2,999	−7.600^***^	−0.221^***^	−9.872 to −5.328	−6.355^***^	−0.185^***^	−8.523 to −4.186	−2.375^*^	−0.069^*^	−4.289 to −0.461
3,000–3,999	−15.227^***^	−0.477^***^	−17.795 to −12.66	−10.729^***^	−0.336^***^	−13.406 to −8.052	−0.728	−0.023	−3.386 to 1.931
≥4,000	−15.599^***^	−0.554^***^	−18.573 to −12.626	−10.587^***^	−0.376^***^	−13.666 to −7.507	−1.319	−0.047	−4.226 to 1.587
**Living arrangement**									
(reference category: Living with spouse and children)									
Living with spouse	−0.873	−0.04	−2.941 to 1.195	−0.451	−0.021	−2.406 to 1.505	−0.646	−0.030	−2.298 to 1.006
Living with children	−0.771	−0.021	−6.641 to 5.098	−2.093	−0.057	−7.646 to 3.460	−4.284	−0.118	−8.985 to 0.417
Living alone	5.526	0.206	−0.835 to 11.887	2.460	0.092	−3.596 to 8.516	0.074	0.003	−5.053 to 5.201
Living with others	−7.02	−0.058	−16.789 to 2.750	−4.934	−0.041	−14.175 to 4.306	−1.223	−0.010	−9.047 to 6.600
Health insurance	−5.127	−0.06	−10.403 to 0.148	−5.886^*^	−0.069^*^	−10.871 to −0.900	−3.625	−0.042	−7.849 to 0.599
Presence of chronic illness(es)									
(reference category: 0)									
1	0.803	0.037	−1.298 to 2.904	0.860	0.040	−1.124 to 2.844	−0.013	−0.001	−1.694 to 1.667
2	2.137	0.089	−0.126 to 4.401	1.351	0.056	−0.795 to 3.498	−0.175	−0.007	−2.001 to 1.651
≥3	3.573^**^	0.130^**^	1.147 to 5.998	2.848^*^	0.104^*^	0.550 to 5.146	0.170	0.006	−1.807 to 2.147
Social support				−0.387^***^	−0.323^***^	−0.484 to −0.289	−0.183^***^	−0.153^***^	−0.270 to −0.096
**Psychological capital**							−0.447^***^	−0.652^***^	−0.51 to −0.385
*R* ^2^		**0.535**			**0.586**			**0.705**	
Adjusted *R*^2^		**0.517**			**0.569**			**0.692**	
△*R*^2^		**0.535**			**0.051**			**0.119**	

*R*^2^: A statistical measure in a regression model that determines the proportion of variance. Adjusted *R*^2^: A modified version of *R*^2^ that accounts for predictors that are not significant in a regression model. △*R*^2^: The change in *R*^2^ between two equations

* *p* < 0.05;

** *p* < 0.01;

*** *p* < 0.001.

### SEM of the Mediating Role of PsyCap Between Social Support and Self-Neglect

**Figure 1:**
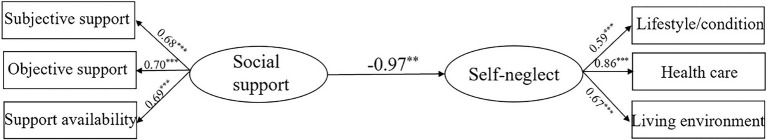
Standardized solution for the structural equation model of social support and self-neglect. ^**^p < 0.01.

**Figure 2:**
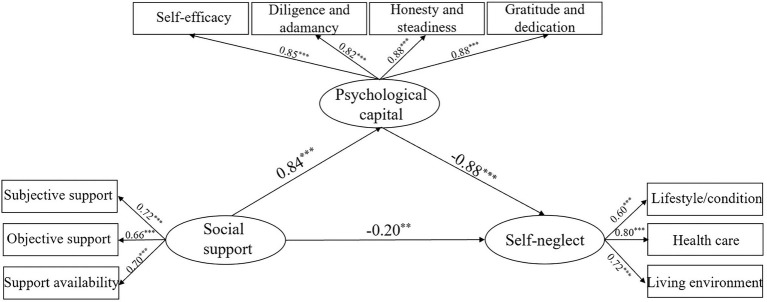
Standardized solution for the structural equation model of psychological capital, social support, and self-neglect. ^**^p < 0.01 and ^***^p < 0.001.

To further confirm the mediating effect of PsyCap between social support and self-neglect, SEM was conducted, with results shown in Table 4. A good fit of the model with obtained data reveals that social support not only directly influences self-neglect, but has a significant indirect effect on self-neglect *via* PsyCap. Figure 1 details the direct path from social support to self-neglect. As hypothesized, social support had a negative and direct impact on self-neglect (*β* = −0.97, *p* < 0.01). The model fits the data well (*χ*^2^/df = 2.74, *p* < 0.05; GFI = 0.991; AGFI = 0.964; IFI = 0.993; CFI = 0.993; TLI = 0.979; RMSEA = 0.058). Figure 2 shows the indirect path from social support to self-neglect mediated by PsyCap (*c* = −0.20, *p* < 0.01), which yielded acceptable goodness-of-fit statistics (*χ*^2^/df = 4.26, *p* < 0.001; GFI = 0.955; AGFI = 0.915; IFI = 0.975; CFI = 0.975; TLI = 0.961; RMSEA = 0.080). As shown, PsyCap was associated with social support (*β* = 0.84, *p* < 0.001) and self-neglect (*β* = −0.88, *p* < 0.001). The path coefficient between social support and self-neglect significantly decreased when PsyCap was added as a mediator (*β* = −0.20, *p* < 0.01). Furthermore, bias-corrected and accelerated bootstrap method demonstrated that PsyCap had indirectly mediated the association between social support and self-neglect (*β* = −0.568, 95% CI: −0.658, −0.474, *p* < 0.001), confirming the medicating role of PsyCap between social support and self-neglect.

**Table 4:** The path coefficients of the mediation model.

	*β*	SE	CR	*P*
Psychological capital ← Social support	0.84	0.162	14.475	<0.001
Self-neglect ← Psychological capital	−0.88	0.052	−10.191	<0.001
Self-neglect ← Social support	−0.20	0.121	−2.769	0.006

*β*, the standardized path coefficient; SE, the standard error; CR, the critical ratio.

## Discussion

This study is the first to explore the relationship between social support, PsyCap, and self-neglect in older adults, and to test the mediating role of PsyCap in the relationship between social support and self-neglect. The correlations between social support, PsyCap, and self-neglect were significant. Additionally, the structural model demonstrated that social support has a negative effect on self-neglect but a positive effect on PsyCap. Further, PsyCap has a negative effect on self-neglect. Our finding supports the hypothesized model that social support negatively influences self-neglect through PsyCap among older adults.

In this study, social support demonstrated a negative association with self-neglect, which is consistent with previous studies. A prior Chinese study reported that older adults with disabilities who received more social support had a lower level of self-neglect and a more stable psychological state (Dong and Sun, 2021). A qualitative study revealed nurses’ perception that limited social support exacerbates the severity of self-neglect (Wu et al., 2020). Decreased mobility because of disease or aging perpetually limits older adults’ ability to maintain their environment and seek treatment or care (Pavlou and Lachs, 2008). Without timely assistance from families and neighbors, self-neglect may be reinforced in case of difficulty (Wu et al., 2020). In China, the children of older adults often struggle with their own work-related responsibilities and spend less time caring for their parents. Increasingly, older adults migrate to cities where they have little interaction with neighbors, resulting in limited support (Yu et al., 2021). This poor social support may further increase older adults’ sense of isolation and neglect (Al Ghassani and Rababa, 2021). Over time, they may doubt their worth, believe they are a burden on society, and feel shame and guilt over asking for help (Burnett et al., 2006; Wu et al., 2020).

A positive relationship was found between social support and PsyCap, which is consistent with prior studies. Mishra and Shafiq reported that social support was positively associated with PsyCap (*r* = 0.37, *p* < 0.01; Mishra and Shafiq, 2018). Gu et al. (2021) found that social support had a positive effect on PsyCap, and PsyCap mediated the relationship between social support and treatment burden in Chinese older patients with chronic obstructive pulmonary disease (Gu et al., 2021). It is possible that material or emotional support, an external positive event, can help older adults preserve existing resources and increase internal psychological resources (Ren and Ji, 2019). Furthermore, social support from family, friends, and the neighborhood has been shown to enhance older adults’ self-efficacy for health-promoting behaviors (Wu and Sheng, 2019) and ultimately improve the status of PsyCap. In general, direct and indirect effects of social support on PsyCap have been evidenced in practice, as detailed above.

This study also revealed a negative correlation between PsyCap and self-neglect. The HMR analysis demonstrated that PsyCap was a protective factor against severe self-neglect. In addition, according to the SEM analysis, PsyCap was found to have a mediating role in the relationship between social support and self-neglect; the negative effect of social support on self-neglect weakened with the mediation of PsyCap, indicating that social support can reduce self-neglect *via* effective control and increase in PsyCap. When living in an environment with stress, difficulties, and limited social and material resources, PsyCap is a protective factor that facilitates active coping behavior and psychological processes (Zhang et al., 2019; Yang et al., 2020). Previous studies have found that PsyCap is closely related to strengthened resilience, psychological wellbeing, and life satisfaction (Pramanik and Biswal, 2020), and those who embrace higher PsyCap are less likely to experience negative psychological effects such as anxiety, depression, and learned helplessness in adversity (Dixon and Frolova, 2011). In Chinese culture, self-actualization, a component of PsyCap, emphasis self-worth and endeavor, which might also play a role in mitigating self-neglect (Shi, 2013; Stodolska et al., 2020). All in all, the present study’s results indicate that the promotion and enhancement of PsyCap might be crucial for reducing or eliminating older adults’ self-neglect.

### Theoretical Significance

This study makes several theoretical contributions. First, it expands our understanding of the potential mechanisms of self-neglect by providing evidence of the key role of PsyCap between social support and self-neglect, which further refines the self-neglect conceptual model proposed by Dyer et al. (2007) and Iris et al.’s (2010) model. Second, a novel model was constructed from the perspectives of both society and positive psychology, providing a basis for building a multidisciplinary and comprehensive theory of handling self-neglect.

### Practical Implications

The study shows that social support and PsyCap affect self-neglect among Chinese older adults, which is meaningful to the process of improving policies and community-based services. On one hand, the government should pay more attention to older adults, with interventions such as investing more to assist older adults with decreased mobility and establish a PsyCap-related cultural atmosphere like “self-actualization is important for the entire society: children and older adults. Caring for children is not everything.” Opportunities for self-actualization are also needed. On the other hand, health workers in the community should not only help older adults increase social contact and address maladaptive social cognition, but also focus on their mental health. To avoid refusal of self-neglect interventions, PsyCap interventions should be undertaken given their recognition in Chinese culture. Family members should also be encouraged to pay attention to older adults’ psychological health, especially in Asian countries where filial piety is practiced.

### Limitations

Although the present study is the first to elucidate PsyCap’s mediating role in the relationship between social support and self-neglect, several limitations should be noted. First, owing to the cross-sectional nature of this study, conclusions cannot be made about causality between variables. Further prospective longitudinal studies are necessary to confirm and build on these findings. The second limitation is that the self-reported questionnaire could be affected by both social desirability bias and shared-method variance. Therefore, future studies are needed to verify the accuracy of the self-reported method.

## Conclusion

Self-neglect among older adults is a rising problem in China. In this study, we found that social support and PsyCap reduced self-neglect, and PsyCap mediated the relationship between social support and self-neglect. Effective interventions should be established to help increase older adults’ social support and PsyCap, which may help promote a positive and stable psychological state, reduce self-neglect, and, more importantly, improve quality of life.

## Data Availability Statement

The raw data supporting the conclusions of this article will be made available by the authors, without undue reservation.

## Ethics Statement

The studies involving human participants were reviewed and approved by Hangzhou Normal University. The patients/participants provided their written or verbal informed consent to participate in this study.

## Author Contributions

BZ: methodology, software, and writing—original draft. HW: investigation. CX: writing—original draft. XH: formal analysis. MC: revision. All authors contributed to the article and approved the submitted version.

## Funding

This work was supported by the Zhejiang Province Philosophy and Social Science Planning Project (19NDJC053YB), Projects of the National Social Science Foundation of China (19BSH034), and Zhejiang Public Welfare Project Fund (LGF20G030007).

## Conflict of Interest

The authors declare that the research was conducted in the absence of any commercial or financial relationships that could be construed as a potential conflict of interest.

## Publisher’s Note

All claims expressed in this article are solely those of the authors and do not necessarily represent those of their affiliated organizations, or those of the publisher, the editors and the reviewers. Any product that may be evaluated in this article, or claim that may be made by its manufacturer, is not guaranteed or endorsed by the publisher.
